# Plasma *miR-21* expression: an indicator for the severity of Type 2 diabetes with diabetic retinopathy

**DOI:** 10.1042/BSR20160589

**Published:** 2017-03-27

**Authors:** Qi Jiang, Xue-Man Lyu, Yi Yuan, Ling Wang

**Affiliations:** 1 Department of Nephrology, China-Japan Union Hospital of Jilin University, Changchun 130033, P.R. China; 2 Department of Ophthalmology, China-Japan Union Hospital of Jilin University, Changchun 130033, P.R. China

**Keywords:** Background diabetic retinopathy, Diabetic retinopathy, Linear regression, Plasma microRNA-21, Proliferative diabetic retinopathy, Type 2 diabetes

## Abstract

To investigate the roles of plasma *miR-21* in the pathogenic process of Type 2 diabetes (T2D) with diabetic retinopathy (DR). T2D patients included patients without DR (NDR) group, patients with non-proliferative/background DR (BDR) group and patients with proliferative DR (PDR) group. Healthy individuals served as control group. Fasting plasma glucose (FPG), glycosylated haemoglobin (HbA1c), triacylglycerol (TG), total cholesterol (TC), urine creatinine (Cr), fasting blood glucose (FBG), blood urea nitrogen (BUN), low-density lipoprotein cholesterol (LDL-C), fasting insulin (FINS) and plasma *miR-21* expression were measured. Quantitative real-time PCR (qRT-PCR) was applied to detect *miR-21* expression. Pearson analysis was used to conduct correlation analysis and receiver operating characteristic (ROC) curve was used to analyse the diagnostic value of *miR-21* in T2D with DR. Compared with the control group, FBG and HbA1c increased in the NDR group; compared with the control and NDR groups, disease course, HbA1c, FPG levels and homoeostasis model assessment of insulin resistance (HOMA-IR) were increased in the BDR and PDR groups; and compared with the BDR group, disease course, HbA1c and FPG levels were higher in the PDR group. *miR-21* expression was higher in the BDR group than the control group, and higher in the PDR group than the BDR group. *miR-21* expression was positively related with disease course, HbA1C, FPG and HOMA-IR, and had diagnostic value for T2D with DR and PDR. The plasma *miR-21* expression was increased in the development of T2D with DR and can be used as an indicator for the severity of T2D with DR.

## Introduction

More than 6% of the world’s population is affected by Type 2 diabetes (T2D) and the prevalence across worldwide is estimated to double by 2025 [1]. Diabetic retinopathy (DR) is one of the major complications in diabetic patients and considered as the major cause of new-onset blindness [2]. Hyperglycaemia promotes vascular damage in T2D patients and the presence of DR is associated with an over 2-fold higher risk of coronary events [3]. DR remains the most common cause of blindness among people aged 30–69 years in several countries [4]. The prevalence of DR ranges from 15.3 to 42.4% in different epidemiologic studies and both modifiable risk factors (blood glucose, pressure and lipids) and non-modifiable risk factors (duration, age and genetic predisposition) are responsible for the progression of DR [5]. Proliferative DR (PDR) is a serious diabetic microvascular complication and a leading cause of visual loss affecting the quality of life of diabetic patients, and neovascularization plays a pivotal role in the development of PDR [6].

*miR-21* is an important member of miRNAs and located on chromosome 17q23-2 overlapping with the *TMEM49* gene [7]. *miR-21* played a critical role in the development of tumours including cell proliferation, migration and metastasis, angiogenesis and anti-apoptosis via a variety of molecular mechanisms [8,9]. *miR-21* is involved in the development of the endocrine pancreas and in the regulation of insulin secretion, and may contribute to β-cell dysfunction in T2D [10]. Besides, miRNAs also implicated in various physiological and pathophysiological processes, including glucose homoeostasis, angiogenesis, inflammatory response modulation and the pathogenesis of diabetes and the related micro- and macrovascular complications [11]. *miR-21* inhibition might be an effective treatment for diabetic nephropathy [12]. However, insufficient data demonstrated the role of *miR-21* in T2D patients with DR.

In the present study, we aim to investigate the role of plasma *miR-21* in the pathogenic process of T2D with DR, especially in diagnosing the severity of DR in T2D. Our study might provide a valuable reference for the clinical diagnosis of T2D patients with DR.

## Materials and methods

### Study subjects

A total of 189 patients diagnosed with T2D were selected from outpatient service and inpatient of endocrinology department during May 2013 and March 2015, including 94 cases of males and 95 cases of females with an age range from 20 to 80 years and a disease course of 0.2 to 24.5 years. The diagnosis was in line with the diagnostic criteria and classification of diabetes of World Health Organization (WHO) in 1999 [13]. All the patients were subjected to a detailed medical examination and fundus ophthalmoscope examination by using a Canon non-mydriasis retinal camera (Canon, Tokyo, Japan). According to the designated staging criteria in the fundi disease academic conference in 2002 [14], T2D patients were divided into three groups: 65 cases without DR (NDR) group including 34 males and 31 females with a mean age of 47.76 ± 8.05 years and a mean duration of 4.16 ± 1.31 years; 73 cases with non-proliferative/background DR (BDR/NPDR) group including 32 males and 41 females with a mean age of 48.83 ± 7.14 years and a mean duration of 9.13 ± 3.05 years; and 51 cases with PDR group including 28 males and 23 females with a mean age of 50.75 ± 10.18 years and a mean duration of 13.58 ± 3.82 years. Exclusion criteria were as follows: patients accompanied by diabetic ketoacidosis, diabetic hyperosmolar coma and other acute complications of diabetes; patients in severe stress such as recent cardiovascular events, trauma surgery etc.; patient suffering from acute or chronic infection; patients with liver disease; and patients combined with other endocrine and metabolic diseases.

A total of 115 non-T2D healthy individuals (60 males and 55 females) were enrolled as control group with matched age and gender proportions and body mass index (BMI) and a mean age of 48.53 ± 7.26 years. An oral glucose tolerance test confirmed that the healthy individuals had no diabetes and a healthy examination excluded the control cases from high blood pressure, heart, liver, kidney diseases and pituitary, thyroid and other endocrine and metabolic diseases. The Canon non-mydriasis retinal camera fundus ophthalmoscope examination revealed no intraocular diseases in the healthy controls. All the cases or their families signed an informed consent form and the experiment was approved by China-Japan Union Hospital of Jilin University and complied with the guidelines and principles of the Declaration of Helsinki [15].

### Data collection

When the patients were admitted, their height and weight were measured and BMI was calculated as weight/height^2^ (kg/m^2^). The disease history, age, gender, disease course of diabetes, glycaemic control etc. were recorded.

### Plasma separation and laboratory testing

All the selected subjects were fasted for 8–12 h. Approximately 6 ml of venous blood sample was extracted at 6:00 the next morning in an EDTA anticoagulant tube at room temperature. The venous blood sample was centrifuged at 3000 rev/min for 10 min. The upper supernatant was taken and sub-packaged in different Eppendorf (EP) tubes, and the sub-packaged plasma was frozen at −80°C for the standby use. Fasting plasma glucose (FPG) was detected by glucose oxidase; triacylglycerol (TG) and total cholesterol (TC) were measured by an enzymatic colorimetric test (GPO-PAP method); glycosylated haemoglobin (HbA1c), blood urea nitrogen (BUN), creatinine (Cr), high-density lipoprotein cholesterol (HDL-C), low-density lipoprotein cholesterol (LDL-C) and fasting insulin (FINS) levels were detected by a Beckman automatic biochemical analyzer (Roche, Germany). Homoeostasis model assessment of insulin resistance (HOMA-IR) was consequently calculated. All steps were in strict accordance with the kit instructions.

### Quantitative real-time PCR

The total plasma RNA of patients was extracted according to kit instructions (Qiagen Company, Valencia, CA). After extraction, the optical density (OD) 260 and OD280 values of each RNA sample were measured using a spectrophotometer (Thermo Fisher Scientific, San Jose, CA). The RNA purity was calculated as OD260/OD280 and RNA concentration was calculated. The purity of RNA was ensured between 1.8 and 2.0. The extracted RNA was preserved at −80°C for use. The cDNA was conducted according to the kit instructions (Qiagen Company, Valencia, CA). According to the gene sequences in the GenBank database and miRBase database, primer 5.0 software was used to design primers. The specific primers with stem–loop structures for *miR-21* and U6 were as follows: *miR-21*: forward: 5'-AAAGGATCCGCCATAGAAACCCAGTTTC-3' and reverse: 5'-GTGCAGGGTCCGAGGT-3'; U6: forward: 5'-CTCGCTTCGGCAGCACA-3' and reverse: 5'-AACGCTTCACGAATTTGCGT-3'. The primers were designed and synthesized by Shanghai Sangon Company (Shanghai, China). PCR reaction system (20 μl): 10 μl of SYBR Premix Ex Taq II (2×), 0.4 μl of forward primer, 0.4 μl of reverse primer, 0.4 μl of ROX Reference Dye II, 2 μl of DNA template and 6.8 μl of dH_2_O. The reaction conditions were 45 cycles of 95°C for 5 s and 60°C for 30 s. U6 was used as an internal control. Melting curves were used to evaluate the reliability of PCR results. *C*_t_ value (amplification power curve inflection) was recorded: Δ*C*_t_ = *C*_t__*miR-21*_ − *C*_t__U6_, ΔΔ*C*_t_ = (*C*_t__*miR-21*_ − *C*_t__U6_)_experimental group_ − (*C*_t__*miR-21*_ − *C*_t__U6_)_control group_ and 2^−ΔΔ*C*_t_^ was used to calculate the relative expression of the target gene. The high expression was defined as the relative expression of *miR-21* larger than the mean value and the low expression was defined as the relative expression of *miR-21* smaller than the mean value.

### Statistical analysis

SPSS 21.0 software (SPSS Inc., Chiago, IL) was used for statistical analysis and measurement data were expressed as mean ± standard deviation. Comparisons between two groups were conducted using *t*-test. Correlation analysis was conducted using Pearson correlation analysis. Multiple linear regression analysis (two-sided α = 0.05) was conducted to analyse the main factors influencing the expression of *miR-21* with related indicators as independent variables. A receiver operating characteristic (ROC) curve was used to analyse the diagnostic value of *miR-21* in T2D with DR. *P*<0.05 was considered statistically significant.

## Results

### Comparisons of general data and laboratory indicators of patients in each group

No significant difference was observed in the general data (except for disease course, fasting blood glucose (FBG) and HbA1c between the NDR group and the control group (all *P>*0.05). Besides, there were no significant difference in gender, age, BMI, FBG, Cr, BUN, FINS, TC, TG and LDL-C levels among the NDR, BDR, PDR and control groups (all *P>*0.05). Disease course, HbA1c, FPG levels and HOMA-IR levels were significantly increased in the NDR group compared with those in the control group (all *P*<0.05). Disease course, HbA1c and FPG were significantly increased in the PDR group compared with the BDR group (all *P*<0.05), showing a gradually increasing trend from the NDR group to the BDR group to the PDR group (Table 1).

**Table 1:** Comparisons of general data and laboratory indicators of patients in each group (*x* ± S.D.)

Group	Gender (male/ female)	Age (year)	Disease course (year)	BMI (kg/m^2^)	FBG (mmol/l)	HbA1c (%)	TC (mmol/l)	LDL-C (mmol/l)	FPG (mmol/l)	TG (mmol/l)	BUN (mmol/l)	FINS (mIU/l)	Cr (μmol/l)	HOMA-IR
Control	60/55	48.53 ± 7.26	/	22.82 ± 2.33	4.74 ± 0.75	4.30 ± 0.42	4.13 ± 0.88	3.21 ± 0.55	5.02 ± 0.68	1.45 ± 0.44	4.89 ± 1.17	5.50 ± 0.88	58.82 ± 7.65	1.25 ± 0.37
NDR	34/31	47.76 ± 8.05	4.16 ± 1.21*	22.67 ± 1.98	7.12 ± 0.68*	6.25 ± 0.58*	4.25 ± 1.02	3.26 ± 0.61	5.31 ± 0.73	1.48 ± 0.32	5.26 ± 1.34	5.66 ± 1.02	59.07 ± 7.88	1.37 ± 0.43
BDR	32/41	48.83 ± 7.13	9.13 ± 2.54*^†^	23.06 ± 1.85	7.25 ± 0.84*	7.02 ± 0.66*^†^	4.32 ± 0.94	3.30 ± 0.63	6.17 ± 0.75*^†^	1.51 ± 0.34	5.33 ± 1.38	5.71 ± 1.14	61.55 ± 8.39	1.60 ± 0.50*^†^
PDR	28/23	50.75 ± 10.18	13.58 ± 3.82*^†^^‡^	23.13 ± 2.02	7.33 ± 1.05*	8.16 ± 0.79*^†^^‡^	4.43 ± 1.08	3.38 ± 0.72	6.86 ± 0.96*^†^^‡^	1.55 ± 0.45	5.41 ± 1.71	5.78 ± 1.21	62.43 ± 10.06	1.81 ± 0.62*^†^

*, compared with the control group, *P*<0.05; ^†^, compared with the NDR group, *P*<0.05; ^‡^, compared with the BDR group, *P*<0.05.

### Comparisons of plasma *miR-21* expression of patients in each group

**Figure 1:**
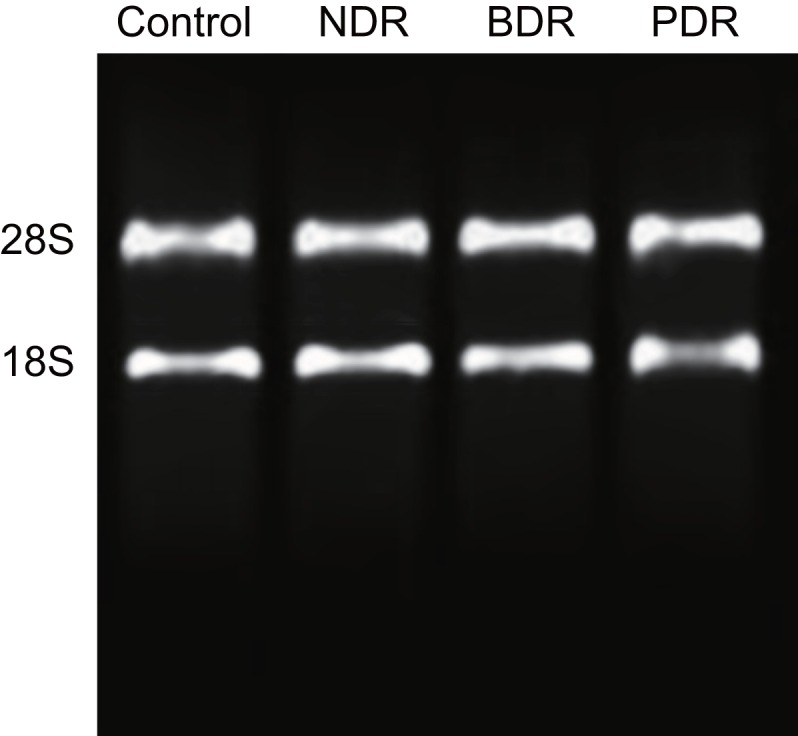
Expressions of plasma *miR-21* of patients in each group

Agarose gel electrophoresis showed that total RNA was successfully extracted from the control, NDR, BDR and PDR groups (Figure 1) and the results of quantitative real-time PCR (qRT-PCR) reaction showed that the *C*_t_ values of the amplification curve were all less than 32, indicating that the experimental data were valid.

The melting curves were all with single peak, indicating good reaction specificity without the interference factors such as non-specific amplification and primer dimers. 2^−ΔΔ*C*^_t_ relative quantification method was used and the relative expression of *miR-21* in the control group was set as 1. Compared with the control group, the *miR-21* expression in the NDR group (1.06 ± 0.01) showed no significant increase (*P>*0.05) and the *miR-21* expression was significantly increased in the BDR and PDR groups (both *P*<0.05). Besides, the *miR-21* expression in the PDR group was significantly higher than those in the BDR group (*P*<0.05) (Table 2).

**Table 2:** Comparisons of expression of plasma *miR-21* of patients in each group

Variable	Control	NDR	BDR	PDR
*C*_t__U6_	27.05 ± 0.21	27.01 ± 0.17	27.36 ± 0.15	27.38 ± 0.16
*C*_t__*miR-21*_	29.27 ± 0.16	29.15 ± 0.18	28.82 ± 0.23*^†^	28.63 ± 0.20*^†^^‡^
2^−ΔΔ*C*^_t_	1.00	1.06 ± 0.01	1.70 ± 0.09*^†^	1.96 ± 0.05*^†^^‡^

*, compared with the control group, *P*<0.05; ^†^, compared with the NDR group, *P*<0.05; ^‡^, compared with the BDR group, *P* < 0.05.

### Correlation between plasma *miR-21* expression and indicators of patients in each group

Pearson correlation analysis showed that the *miR-21* expression in each group was positively correlated with disease course (*r*=0.794), HOMA-IR (*r*=0.326), HbA1c (*r*=0.693) and FPG (*r*=0.598) (all *P*<0.001). However, there was no significant correlation between *miR-21* and TG, TC, Cr, BUN, HDL-C, FINS or LDL-C (all *P>*0.05) (Table 3).

**Table 3:** Correlation between expression of plasma *miR-21* and indicators of patients in each group

Indicators	Disease course	TG	TC	LDL-C	HbA1C	Cr	BUN	FINS	FPG	HOMA-IR
*r*	0.794	0.082	0.076	0.078	0.693	0.134	0.051	0.052	0.598	0.326
*P*	<0.001	0.265	0.296	0.285	<0.001	0.065	0.486	0.478	<0.001	<0.001

Cr, creatinine.

### Multivariate linear regression analysis of *miR-21*-related factors

Multiple linear regression analysis showed that the disease course, HbA1c, FPG and HOMA-IR were all main factors influencing the plasma *miR-21* expression (all *P*<0.05). The regression equation was as follows: *Y* = 1.424 + 0.02*X*_1_ + 0.74*X*_2_ + 0.21*X*_3_ − 0.413*X*_4_ (*X*_1_, disease course; *X*_2_, FPG; *X*_3_, HOMA-IR; *X*_4_, HbA1c; *F*=13.727, *P*<0.001). Multiple correlation coefficient *R*_2_=0.379, and the independent variables of the equation and related parameter values were shown in Table 4.

**Table 4:** Multivariate linear regression analysis of *miR-21*-related factors

Variable	Regression coefficient	S.E.M.	*B*	*t*	*P*
Constant	1.424	0.413	–	3.450	0.001
Disease course	0.020	0.006	0.245	3.535	0.001
FPG	0.740	0.206	1.975	3.594	<0.001
HOMA-IR	0.210	0.068	0.300	3.107	0.002
HbA1c	−0.413	0.159	−1.097	−2.597	0.010

*B*, standardized regression coefficient.

### Diagnostic value of *miR-21* in DR

**Figure 2:**
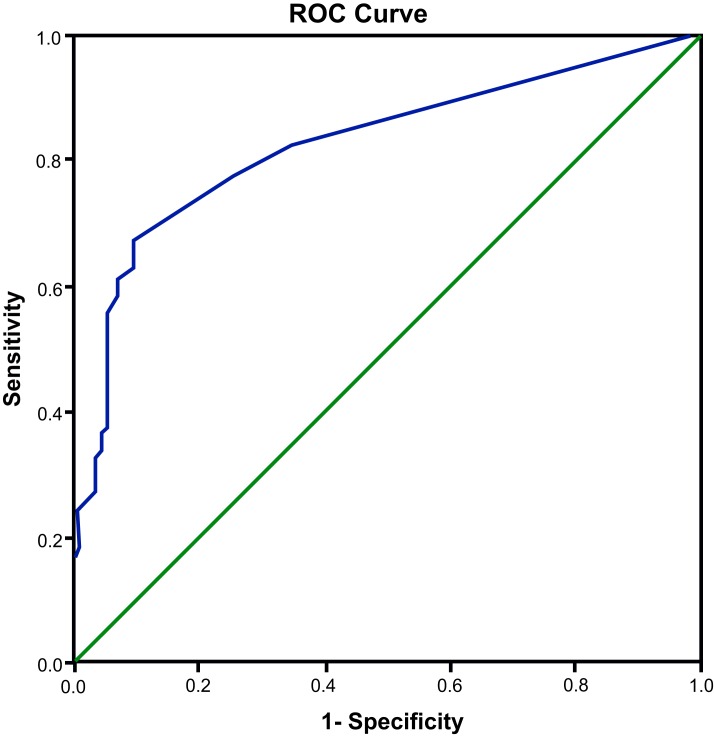
ROC curve of plasma *miR-21* in diagnosing DR

ROC curve analysis was applied to measure the diagnostic value of *miR-21* in DR with the control and NDR groups (−) and the BDR and PDR groups (+) as the two categorical variables. The results showed an area under the curve (AUC) of 0.825 (95% confidence interval (95% CI) = 0.778−0.872; *P*<0.001), a sensitivity of 66.1% and a specificity of 90.4%, which indicated that plasma *miR-21* expression had a relative predictive value for T2D with DR (Figure 2).

### Diagnostic value of *miR-21* in PDR

**Figure 3:**
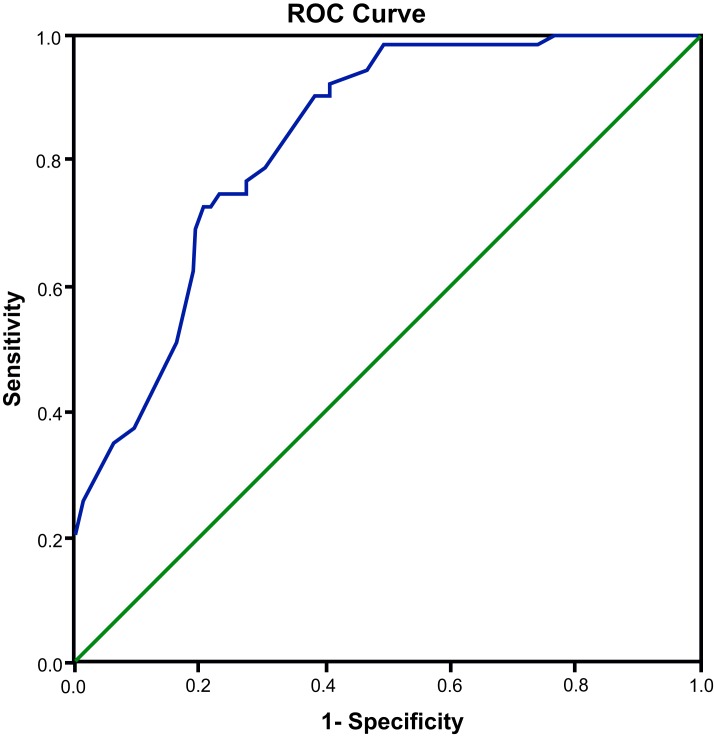
ROC curve of plasma *miR-21* in diagnosing PDR

ROC curve analysis was applied to measure the diagnostic value of *miR-21* in PDR. The results showed that using *miR-21* to diagnose PDR yield an AUC of 0.830 (95% CI = 0.761−0.900; *P*<0.001), with a sensitivity of 72.5% and a specificity of 79.5%, indicating that plasma *miR-21* expression had a relative predictive value for PDR (Figure 3).

## Discussion

In the present study, we investigated the role of plasma *miR-21* in the pathogenic process of T2D with DR. One of our main results showed that compared with the control group, the *miR-21* expression was relatively increased in the BDR and PDR groups. Besides, the *miR-21* expression in the PDR group was significantly higher than that in the BDR group. The results indicated that *miR-21* was associated with the pathogenic process of DR in T2D and the severity of DR. Aberrant miRNA expression profiles were associated with the DR development, and modulation of retinal miRNA expression may provide a potential treatment for DR [16]. The *miR-126* was associated with sight-threatening DR, suggesting that the *miR-126* would be a potential therapeutic target for inhibiting DR development [17]. *MiR-23b-3p* regulates high glucose-induced cellular metabolic memory in DR via a SIRT1-dependent signalling pathway [18]. The expressions of several miRNAs related to angiogenesis and fibrosis were expressed significantly higher in PDR [19].

As for the mechanism of *miR-21* in the development of T2D with DR, we investigated the relationships between *miR-21* and some clinical indicators. Our correlation analysis showed that *miR-21* expression was positively correlated with disease course, HbA1c, FPG and HOMA-IR. Besides, the multiple linear regression analysis confirmed that the disease course, HbA1c, FPG and HOMA-IR were all main factors influencing the plasma *miR-21* expression. HOMA-IR is a robust tool for the assessment of insulin resistance (IR) and is most widely used in a large population [20]. Moderate and large increases in HOMA-IR had a strong impact on the development of T2D with impaired insulin secretion [21]. HbA1c monitoring is often needed for patients with variable glycaemic control and receiving intensive insulin treatment, and is used as a diagnostic marker for T2D and T2D associated complications [22,23]. The HbA1c variability increases to the risk of DR independently of average metabolic control [24]. A threshold of an FPG of 7.0 mmol/l defines the presence of diabetes provided by the rationale for American Diabetes Association’s recommendation and DR has served as the basis for diagnostic criteria of T2D [25].

Another important result of our study showed that plasma *miR-21* level had a relative predictive value for the existence and the severity of T2D with DR. DR was an ocular disease and governed by systemic and local ocular factors, including primarily chronic levels of blood glucose [26]. DR results in progressive visual deterioration from mild, moderate, severe to very severe NPDR with a risk of developing PDR [27]. NPDR is a first stage of DR, marked by gradual capillary dropout, and associated with a set of clinically observable changes in the microcirculation, which are focal disruptions of the capillary topology [28]. PDR is a serious T2D complication of diabetes and a leading cause of legal blindness and visual impairment [29]. Combination of *miR-21*, *miR-181c* and *miR-1179* had a moderate ability to discriminate between PDR and NPDR and the accuracy rate of the three miRNA profiles was 82.6%, suggesting that serum miRNAs had the potential to be sensitive, cost-effective biomarkers for PDR detection from NPDR [30].

Taken together, our results demonstrated that plasma *miR-21* expression was increased in the development of DR and can be used as an indicator for the severity of DR in T2D patients. The role of *miR-21* in the development of T2D with DR was related with disease course, HbA1c, FPG and HOMA-IR. Our study provided valuable references for diagnosis of T2D with BDR and PDR. However, a clear description of the relationship between *miR-21* and T2D with DR is needed more data to support the relative importance of each part. It is likely that deepening our understanding in this field, and hopefully, more experiments to prove evidence for the correlation between miRNAs and T2D with DR are needed in the future.

## Abbreviations

AUC: area under the curve
BDR: T2D patients with background DR group
BMI: body mass index
BUN: blood urea nitrogen
CI: confidence interval
Cr: creatinine
DR: diabetic retinopathy
FBG: fasting blood glucose
FINS: fasting insulin
FPG: fasting plasma glucose
GPO: glycerophosphate-oxidase
HbA1c: glycosylated haemoglobin
HDL-C: high-density lipoprotein cholesterol
HOMA-IR: homoeostasis model assessment of insulin resistance
IR: insulin resistance
LDL-C: low-density lipoprotein cholesterol
NDR: T2D patients without DR
NPDR: T2D patients with non-proliferative DR
OD: optical density
PDR: T2D patients with proliferative DR
qRT-PCR: quantitative real-time PCR
SYBR: synergy brands
TC: total cholesterol
T2D: Type 2 diabetes
TG: triacylglycerol
TMEM: transmembrane protein 4
